# Study design and protocol for a mixed methods evaluation of an intervention to reduce and break up sitting time in primary school classrooms in the UK: The CLASS PAL (Physically Active Learning) Programme

**DOI:** 10.1136/bmjopen-2017-019428

**Published:** 2017-11-08

**Authors:** Ash C Routen, Stuart J H Biddle, Danielle H Bodicoat, Lorraine Cale, Stacy Clemes, Charlotte L Edwardson, Cris Glazebrook, Deirdre M Harrington, Kamlesh Khunti, Natalie Pearson, Jo Salmon, Lauren B Sherar

**Affiliations:** 1 School of Sport, Exercise and Health Sciences, Loughborough University, Loughborough, UK; 2 Active Living & Public Health, Institute of Sport, Exercise and Active Living (ISEAL), Victoria University, Melbourne, Victoria, Australia; 3 Institute for Resilient Regions, University of Southern Queensland, Springfield, Queensland, Australia; 4 Diabetes Research Centre, University of Leicester, Leicester, UK; 5 The Leicester Biomedical Research Centre, Leicester and Loughborough, UK; 6 School of Medicine, Division of Psychiatry and Applied Psychology, University of Nottingham, Nottingham, UK; 7 Institute for Physical Activity and Nutrition (IPAN), School of Exercise and Nutrition Sciences, Deakin University, Geelong, Australia

**Keywords:** physical activity, classroom, school, intervention, implementation

## Abstract

**Introduction:**

Children engage in a high volume of sitting in school, particularly in the classroom. A number of strategies, such as physically active lessons (termed movement integration (MI)), have been developed to integrate physical activity into this learning environment; however, no single approach is likely to meet the needs of all pupils and teachers. This protocol outlines an implementation study of a primary school-based MI intervention: CLASS PAL (Physically Active Learning) programme. This study aims to (A) determine the degree of implementation of CLASS PAL, (B) identify processes by which teachers and schools implement CLASS PAL and (C) investigate individual (pupil and teacher) level and school-level characteristics associated with implementation of CLASS PAL.

**Methods and analysis:**

The intervention will provide teachers with a professional development workshop and a bespoke teaching resources website. The study will use a single group before-and-after design, strengthened by multiple interim measurements. Six state-funded primary schools will be recruited within Leicestershire, UK.

Evaluation data will be collected prior to implementation and at four discrete time points during implementation: At measurement 0 (October 2016), school, teacher and pupil characteristics will be collected. At measurements 0 and 3 (June–July 2017), accelerometry, cognitive functioning, self-reported sitting and classroom engagement data will be collected. At measurements 1(December 2016–March 2017) and 3, teacher interviews (also at measurement 4; September–October 2017) and pupil focus groups will be conducted, and at measurements 1 and 2 (April–May 2017), classroom observations. Implementation will be captured through website analytics and ongoing teacher completed logs.

**Ethics and dissemination:**

Ethical approval was obtained through the Loughborough University Human Participants Ethics Sub-Committee (Reference number: R16-P115). Findings will be disseminated via practitioner and/or research journals and to relevant regional and national stakeholders through print and online media and dissemination event(s).

**Strengths and limitations of this study**

- Intervention coproduced with schools and teachers.
- Evaluation over whole school year.
- Use of mixed methods to capture implementation information.
- Study sample limited to one school year group.

## Introduction

There is now good evidence to suggest high amounts of sedentary behaviour (ie, sitting/reclining activities during waking hours requiring low energy expenditure^1^) during adulthood pose a distinct risk for a range of negative health outcomes including type 2 diabetes, cardiovascular disease and all-cause mortality, even when accounting for participation in moderate-to-vigorous physical activity (MVPA).^2 3^ Evidence for such associations in children is more limited; however, cross-sectional analyses have shown associations between screen-based sedentary behaviour and lower cardiorespiratory fitness,^4^ increased accelerometer assessed sedentary time and lower HDL cholesterol^5^ and higher fasting insulin.^6^

During weekdays, primary school children spend the majority of their waking hours at school, where between 50% and 70% of time is spent sitting.^7–9^ This has led to calls by some researchers for interventions to change the way children ‘work’ in schools.^8 10^ While time outside of school has been shown to be increasingly sedentary, and more so than during school hours, schools provide easier access and maximum reach for intervention efforts.^11 12^ The potential for exposure to prolonged sitting in the classroom clearly presents a notable opportunity to intervene on children’s daily sitting behaviour by integrating physical activity into normal classroom instruction time (termed movement integration (MI)).

A number of MI interventions have been evaluated for effectiveness in recent years. These include short duration activity breaks (often referred to as ‘fitness breaks’),^13 14^ longer duration activity breaks that incorporate academic content^15^ and more comprehensive physically active academic lessons that deliver curriculum content through movement.^16 17^ A systematic review of 11 MI interventions showed encouraging preliminary evidence of improved daily physical activity and educational outcomes.^18^ Other studies have also shown reductions in classroom sedentary time and small increases in MVPA.^19 20^ Furthermore, more recent randomised controlled trials have shown improvements in academic achievement in some subjects (numeracy and literacy)^21^, particularly for lower achieving students^22^, following participation in physically active lessons. While the review and studies cited above are encouraging and present an optimistic picture in relation to positive outcomes, there is a notable lack of evidence on how to translate these MI interventions into everyday (or routine) classroom practice.

Within the field of public health, there is rapidly growing recognition of a long history of failure to transfer or replicate evidence-based approaches, developed in controlled settings, to the complexity, diversity and dynamism of ‘real world’ settings.^23^ In response, the field of implementation research has developed to study the ‘processes used in the implementation of initiatives as well as the contextual factors that affect these processes'.^24^ Often, however, attempts to study implementation are made via the use of programme theories and expected mechanisms and outcomes, or retrospective process evaluations,^25^ none of which explicitly focus on studying the processes or context of implementation from the outset. For example, process evaluations have largely focused on issues such as fidelity or programme acceptability,^20^ as opposed to identifying factors that contributed to, or conversely constrained, implementation of the intervention. In addition, the majority of physical activity intervention publications over the past three decades have been efficacy trials, and only 3% have included implementation/dissemination studies.^26^ The uniqueness and importance of taking an implementation research approach is that it recognises the need for a range of conceptual and methodological tools and methodologies to understand and outline the complex processes of engagement in real-world interventions from the very outset of the conception of an intervention programme.^27^

An important consideration in implementation research is that of the *context* (setting, roles, interactions and relationships) that is being intervened on and the *plasticity* of the intervention (ie, the degree of flexibility possessed by a set of intervention components).^25^ In relation to the aforementioned MI interventions, these have generally targeted a single type of strategy, as part of a prescribed approach (eg, 3 × active lessons for 10 weeks), thus having low plasticity.^25^ Within the school and teaching context, however, to elicit sustainable changes to classroom teaching practice at population level, teachers will require a more flexible (ie, plastic) intervention approach. For example, whereby they can use MI strategies that fit the particular needs of their class at a given time (eg, using a brief active break to re-engage a group of students who are off-task or to transition from one subject area to another).

The CLASS PAL (Physically Active Learning) programme was based on some of the core principles from previously published Active Learning Trials, namely The Virtual Traveler^28^ and Transform-Us!,^29 30^ and developed in collaboration with a range of school stakeholders to support primary school teachers in the integration of various modes of MI as matter of routine practice. The evaluation of the programme will help to understand implementation processes and further facilitate its development as an MI intervention that is sensitive to individual school contexts and that has inherent plasticity.

### Aim

The purpose of this paper is to outline the protocol for an implementation study of a primary school-based MI intervention: CLASS PAL (Physically Active Learning) programme. The objectives of this study are to: (A) determine the degree of implementation of CLASS PAL, (B) identify the processes by which teachers and schools implement CLASS PAL and (C) investigate the individual (pupil and teacher) level and school level characteristics associated with implementation of CLASS PAL.

## Methods and analyses

### Study design

CLASS PAL is a MI intervention that incorporates a 1-day continuing professional development (CPD) workshop for teachers, alongside a website with a bank of teaching resources (www.classpal.org.uk). Participating teachers will attend the workshop in October 2016, and the study will follow these teachers and their class pupils until the end of the school academic year in July 2017 and teachers alone until the start of the following academic year in September/October 2017. Implementation research needs multimethod approaches to support the considerable complexity of stages and processes.^31^ Thus, it was established that the intervention would be evaluated using a single group before-and-after study, strengthened by multiple additional interim measurements taking a mixed methods approach with information collected on actions, inputs, resources and attitudes required for its successful implementation. Data will be collected at measurement 0 (before the CPD workshop), and then at four time points during the implementation period.

### Recruitment and study participants

Six state-funded primary schools from the county of Leicestershire (UK) will be recruited to participate in the evaluation. Where possible, all year five classes (children aged 9–10 years) and their teachers from each school will participate. Year 5 students were selected because when consulted in the preparatory phase of the intervention, teachers felt that children spent more time sitting as they progressed through primary school, which has also been supported in the literature.^32^ Year 6 students were not considered as the pressures from standarised tests were deemed a challenge when piloting a new teaching style. If a class has mixed year groups (eg, year 5 and year 6 combined), they will still be eligible. Schools will be recruited via a face-to-face meeting with head teachers, facilitated by a Teaching School Alliance^i^. Recruiting via this Alliance enabled strong working relationships to be built with schools during the pilot work for the programme^33^ and recruitment of a more representative sample (approximately 85% of primary schools in Leicestershire are part of an Alliance^ii^, and there are around 600 Alliances nationally^34^). Schools will be asked to provide an initial expression of interest, following which they will be categorised into tertiles based on the Income Deprivation Affecting Children Index,^35^ and then further stratified by geographic location (eg, rural, town and fringe, urban and so on, identified via http://www.education.gov.uk/edubase/home.xhtml) and school size (number of pupils on roll, also identified via EduBase). A minimum of two schools will be selected from each tertile; this will include a range of schools differing in size and geographic location (ie, rural/urban). Consideration will also be given to school’s capacity to take part in the programme; this information will be provided by the Teaching School Alliance.

Following school selection, a telephone call with the head teacher will be arranged to explain the study details further, prior to schools providing formal agreement to participate. After school recruitment, teachers and pupils will then be invited to participate in the evaluation activities of the programme via a letter to parent(s)/guardian(s), parent/guardian information sheet, child information sheet and an opt-out consent form for the parent/guardian (whereby the parent/guardian only returns a signed form if they do not want their child to participate in the evaluation activities). Participating children will also be asked to sign an assent form prior to the first data collection session.

### Intervention

#### Intervention development

CLASS PAL is designed to provide primary school teachers with the training and teaching resources necessary to implement any kind of MI on a regular basis. This could include active routines (eg, ‘Stand up, Hand up’), activity breaks (eg, dancing to YouTube video), activity breaks incorporating academic content (eg, jumping to solve mathematical sums) or more comprehensive physically active academic lessons (eg, physically embodying punctuation marks as teacher reads story aloud). Teachers will be advised to implement activities as a matter of everyday routine, when they see utility and as and where feasible. There will be no prescription regarding the frequency, duration or type of classroom physical activities; however, teachers will be encouraged to set personalised goals. This is designed to replicate how teachers would likely implement MI in a real-world setting.

An initial intervention concept of a professional development workshop and supporting teaching resources was developed from formative qualitative interviews and focus groups with 25 primary school teachers and 10 pupils in Leicestershire. Following this preliminary qualitative work, a coproduction development phase was conducted with a different group of seven teachers from six schools in Leicestershire between April and July 2016.^33^ This process was also facilitated by the Youth Sport Trust,^iii^ who were commissioned to aid in supporting the resource and workshop development and delivery. During this phase, the teachers attended a programme launch at the National Centre for Sport and Exercise Medicine at Loughborough University, where they were introduced to the programme and provided with some brief training and a video lesson capture tool (www.irisconnect.co.uk). Following the launch event, teachers then tested the MI strategies, video recorded them and shared them with the research team and pilot group via a secure website, feeding back on any barriers to implementation as well as their training needs. Concurrently, two further coproduction events were held in the schools to identify barriers to implementation of MI, training needs, and to support the teachers’ resource requirements.

### Behavioural framework

**Figure 1:**
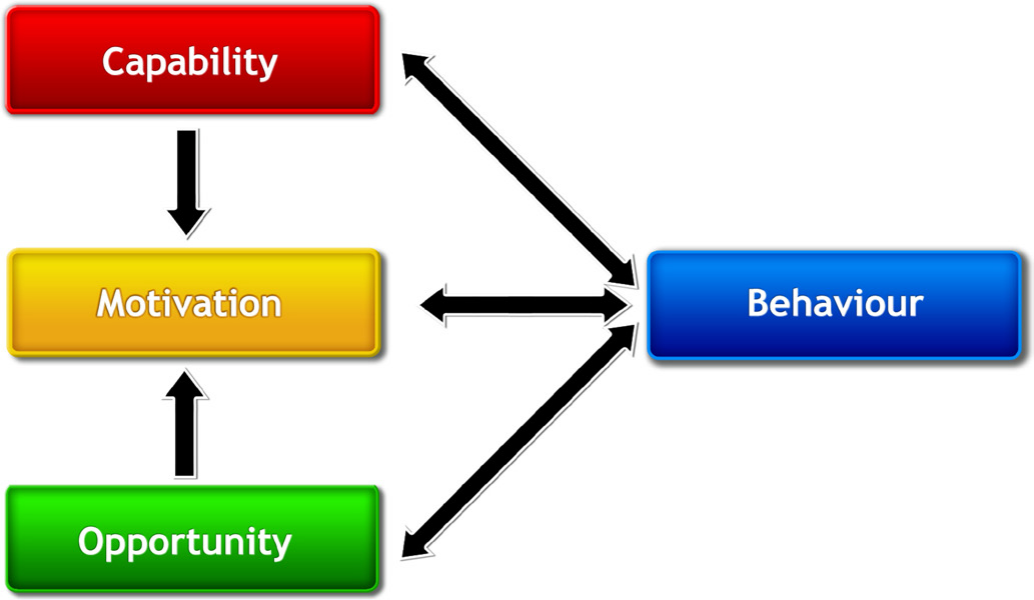
COM-B model of behaviour.

There is evidence that physical activity interventions guided by behavioural theory are more effective and sustainable than those that do not use/specify a theory.^36^ However, a recent systematic review of physically active lesson interventions identified the limited use of underpinning behavioural theory.^18^ Therefore, the ‘co-production’ approach taken to develop the CLASS PAL intervention was guided by a contemporary and overarching model of behavioural theory: the COM-B model^37^ (shown in figure 1). The COM-B behavioural system proposes three sources of behaviour: capability, motivation and opportunity. For CLASS PAL, the agent of change for which these sources must be targeted is the classroom teacher.

Capability is defined as ‘the individual’s psychological and physical capacity to engage in the activity concerned’. It includes having the necessary knowledge and skills. Motivation is defined as ‘all brain processes that energise and direct behaviour, not just goals and conscious decision-making’. It includes habitual processes, emotional responding, as well as analytical decision-making. Opportunity is defined ‘as all the factors that lie outside the individual that make the behaviour possible or prompt it’.^37^

Psychological capability will be addressed in CLASS PAL through professional development training, providing teachers with the necessary knowledge and skills to be able to implement MI. Physical capability will be enhanced through gaining head teacher support for teachers to engage with the programme and have the freedom to alter their teaching practice (and the physical environment if required). Enhancing motivation will be targeted in the teacher training workshop and through the website by providing information on the educational and health benefits of MI and by the provision of a comprehensive set of MI resources through the website. Opportunity will be addressed by ensuring that teaching resources are appropriate/adaptable to accommodate for space and class size constraints and pupils’ needs, with a practical session also included in the workshop to directly address this issue.

Alongside the COM-B model, taxonomies of behaviour change techniques (BCTs) have been developed to identify active intervention ingredients and allow future interventionists the possibility of replication.^38–40^ The BCT that will be used in CLASS PAL are displayed in table 1. The BCT used were based on two prior MI interventions implemented in UK primary schools.^16 28^ These are classified using the Behaviour Change Taxonomy V.1.^40^

**Table 1:** Behaviour change techniques (BCTs) used in the CLASS PAL intervention

Taxonomy Category	BCT	Definition	Example
Shaping knowledge	1. Instruction on how to perform the behaviour	Advise or agree on how to perform the behaviour (includes skills training)	Training at workshop provides information/modelling on how to perform movement integration. Website provides resources/instruction to support implementation.
Natural consequences	2. Information about health/education outcomes/consequences	Provide information about health outcomes/consequences of performing the wanted/unwanted behaviour	Workshop and website provide information on the health disbenefits of prolonged sitting and the benefits of active learning to education and health outcomes.
Antecedents	3. Restructuring the physical environment	To change the physical environment in order to facilitate the performance of the wanted behaviour	Teachers advised in workshop on how to arrange classroom to allow space for movement.
Comparison of behaviour	4. Information about others’ approval	Provide information about what other people think of the behaviour	Videos of teachers’ and pupils’ views from the pilot phase of the project shared with teachers through the website. Teachers from the pilot phase also attending the workshop to share views of implementing movement integration.
Comparison of outcomes	5. Credible source	Present verbal or visual information from a credible source in favour of the behaviour	Peer-reviewed published research on movement integration shared in workshop and on website.
Goals and planning	6. Problem solving	Analyse or prompt the person to analyse factors influencing the behaviour and generate or select strategies that include overcoming barriers and/or increasing facilitators	Teachers prompted to discuss barriers/facilitators to movement integration and to identify solutions.
Feedback and monitoring	7. Enablement	Self-monitoring of behaviour	Teachers asked to keep a log (online or paper/pencil version if preferred) of movement integration delivery including date, time, activity type, resource(s) used, duration of activity and pupil engagement.
Repetition and substitution	8. Behaviour substitution	Prompt substitution of unwanted behaviour with a wanted behaviour	Teachers encouraged at the workshop to implement movement integration to replace previously sedentary time/teaching practices.
Goals and planning	9. Goal setting	Set or agree on a goal defined in terms of the behaviour to be achieved	Teachers complete pledge postcard, with three movement integration goals. To be mailed back to teacher 6 weeks post-workshop by another attendee.

### Professional development workshop

Following measurement 0 measurements, all participating teachers will be required to attend a 1-day professional development workshop that will be held in the Teaching School Alliance training facility and delivered by two former primary school teachers (Youth Sport Trust employees). Schools will be provided with an honorarium of up to £200 to contribute to supply cover to facilitate teachers’ attendance at the workshop.

The first module of the training will begin with a number of activities designed to facilitate reflection on the characteristics of good teaching; the second module will discuss the case for MI from a research and teaching perspective (BCTs 2 and 5; table 1); module three comprises activities focused on overcoming barriers to MI^3 6^; module four will include activities to build understanding of the components of a physically active classroom (ie, changes in routines, activity breaks and physically active lessons). Included in this module is an extended group task where the teachers will develop and model physically active lesson ideas mapped to concepts within the National Curriculum for England.^1 8^ The final workshop module (five) will include an overview of the CLASS PAL website and a sharing, feedback and future planning/goal setting session.^7^ This will include writing three MI goals on a postcard and giving it to another attendee to post back to the individual 6 weeks later.^9^

Email and telephone contact details of the first author (ACR) will be given to all teachers in the event that more support is required for the intervention or evaluation. All additional contact/support will be logged.

### Evaluation procedures

Data collection for the evaluation will take place over 9 months in each of the participating classes. The evaluation measures will be taken at times convenient for the class teacher, and each data collection session will last approximately 2 hours. Data will be collected on five occasions: at measurement 0 (October 2016), measurement 1 (December 2016–March 2017), measurement 2 (April–May 2017), measurement 3 (June–July 2017) and measurement 4 (September–October 2017). Teacher characteristic and school environment data will be collected at measurement 0 only. Pupil questionnaire, anthropometrics (measurement 0 only), objective physical activity data and cognitive functioning data will be collected at measurement 0 and measurement 3 only. At measurement 1 and measurement 2, classroom observations will be conducted, at measurements 1 and 3, pupil focus groups will be conducted and at measurements 1, 3 and 4, teacher interviews will be conducted. During the whole implementation period, teachers will also complete a self-report implementation log and website analytics will be recorded.

### Evaluation framework

There now exists a plethora of theories derived to understand or explain effective implementation, including meta-theories such as the Consolidated Framework for Implementation Research.^41^ The current evaluation approach does not subscribe to a particular theory or framework but, as mentioned earlier, draws on the fundamental tenets of implementation research, which is the scientific study of the processes used to implement policies and interventions and the contextual factors that affect these processes.^24^ Furthermore, the evaluation will focus on five of the eight features of implementation, namely: fidelity of delivery, dosage received, quality, participant responsiveness and adaptation.^27^ Fidelity relating to teacher implementation is considered to be the integrity of MI delivery (ie, delivery includes physical activity in normal classroom time and delivery is supported by resources from CLASS PAL website). Dosage refers to how much MI the teachers have delivered (ie, frequency, intensity and duration). Quality refers to how well MI is delivered by teachers (ie, engaging MI delivered with careful preparation, clear instruction, designed to overcome barriers and so on). Participant responsiveness refers to the degree to which the delivery of MI engages and/or holds the attention of the pupils. Finally, adaptation refers to the degree of modification by teachers of the CLASS PAL resources and MI principles/recommendations for the implementation of MI.

It is also planned to seek to determine the integrity of the delivery of the teacher training workshop in relation to the core aims of the training, as well as the quality and dose of delivery received.

### Measurements and instruments

All self-report data from the teachers and pupils will be collected via paper-based questionnaires administered in the classroom.

### Study objective A: to determine the degree of implementation of CLASS PAL

#### Workshop evaluation (fidelity, dosage and quality)

At the end of the intervention workshop, delegates will complete a paper-based evaluation questionnaire including items on the quality of resources, delivery, content, perception of tutors’ knowledge, workshop duration, adequacy of training, teachers’ confidence to deliver MI and suggestions for future improvements.

#### Teacher log (fidelity and dosage)

Teachers will be asked to complete a log of all delivered MI during the measured implementation period (postworkshop to measurement 3). They will be given a choice to complete this via a hard copy wallpaper chart or via an online survey (www.surveymonkey.co.uk) that can be completed via smartphone, tablet or desktop computer. In addition to recording the date and time of delivery, six questions will be employed to capture information on the type of activity delivered (eg, physically active lesson), supporting resources used (eg, personally developed resource), instruction type (eg, teacher led), duration of the activity and the pupils’ movement response (eg, less than 50% of pupils were active). This will provide data on the frequency and duration (delivery) of activities, as well as the degree of participation (pupil response). These questions have been adapted from the System for Observing Student Movement in Academic Routines and Transitions.^42^

#### Direct observation: classroom on-task behaviour and physical activity (fidelity and dosage)

At measurements 1 and 2, on-task behaviour before, during and after MI will be examined using the Observing Teacher and Pupils in Classrooms tool.^43^ This rates behaviour as on-task (making eye contact with teacher or task and following teacher’ instructions) or off-task. In addition, the Children’s Activity Rating Scale will be used to code the intensity of physical activity behaviour.^44^ This tool codes observed activities into five categories of increasing intensity: stationary, stationary with limb or trunk movements, slow movement, moderate movement and fast movement.^44^ Both observation tools will be used concurrently during observed class periods to reduce the burden on schools and the researcher. One previously agreed lesson where the teacher is intending to implement a physically active lesson (or activity for a significant portion of the lesson) or series of active breaks will be observed. Classes will be observed (using whole time sampling), for a minimum of 10 min prelesson, during the whole lesson and for a minimum of 10 min postlesson. Pupils will be observed (scanning from left to right of room and focusing on child in clear sight and closest to left) for 5 s in turn, before the next pupil is observed and data recorded.^28^ Prior to data collection, an observer training session will be run, and one observer will collect the data. Inter-rater reliability will be calculated by a second observer viewing a subsample of lessons, with acceptable reliability deemed to be 70% agreement.

#### Website usage (fidelity)

From Measurement 0 to Measurement 3, website usage data will be tracked using Google Analytics.^45^ The main outcomes of focus will be the number, depth (ie, additional further page views in the visit) and duration of unique visits to the resources page by the participating teachers (differentiated from other visitors to the site as access to the resources requires input of login details). The traffic source (ie, referral route and content exposure (eg, number of times a particular page served as entrance to the resource section of the website))^45^ will also be documented.

### Study objective B: to identify the processes by which teachers and schools implement CLASS PAL

#### Teacher interview (fidelity, dosage, quality, participant responsiveness and adaptation)

At measurements 1, 3 and 4, teachers will participate in a semistructured interview (face to face or telephone). Interviews will be used to elicit information on the processes of delivery of CLASS PAL, acceptability to the teachers, pupils’ responsiveness and barriers and facilitators to implementation. Interviews will last for approximately 30 min and will be recorded using a digital audio recorder and transcribed verbatim.

#### Pupil focus group (fidelity, dosage, quality and participant responsiveness)

At measurements 1 and 3, a mixed-sex sample of year 5 pupils from each school will participate in semistructured focus groups. Guidelines will be provided to teachers on the selection of pupils to ensure a range in terms of learning style/abilities and character. Questions will explore pupils’ exposure to the intervention, their level of engagement with the intervention and their perceptions of enjoyment, acceptability and dislikes and challenges to participation. Focus groups will last for approximately 40 min, allowing for the building of rapport, and will be recorded using a digital audio recorder and transcribed verbatim.

### Study objective C: to investigate the individual (pupil and teacher) level and school-level characteristics associated with the quantity of implementation of CLASS PAL

#### School

##### School environment

At measurement 0, teachers will complete a school characteristics questionnaire covering the following: availability, opportunities and access to physical activity and recreation facilities; physical activity, Physical Education and sport policies and practices; participation in physical activity or sport initiatives or programmes; and accreditation/award schemes. These questions were adapted for the UK context from the International Study of Childhood Obesity, Lifestyle and the Environment school administrator questionnaire.^46^

##### Classroom environment

At measurement 0, the school floor plan will be requested to ascertain the floor space available for MI in the intervention classrooms. Available floor space will be expressed as metres squared (m^2^). During lesson observations at measurements 1 and 2, photographs/and or sketches and field notes on class layout will also be collected.

#### Teacher

##### Demographic, teaching experience and qualifications

At measurement 0, teachers will be asked to provide their name, date of birth, ethnicity, details of their teaching experience/training and qualifications (using a mixture of select response and open-ended questions). This will include, for example, four specific items on prior training in physical education, health, sport, physical activity and MI.

##### Self-reported physical activity and sedentary behaviour

At measurement 0 and measurement 3, teachers will report on how many days per week they do 30 min of MVPA or more using a single item.^47^ Weekday sedentary behaviour will be assessed using the sitting item from the short version of the International Physical Activity Questionnaire.^48^

##### MI: current use and competence​

At measurement 0 and measurement 3, teachers will report how often they integrate movement into the classroom using a series of six items from the Physical Activity Promotion in the Academic Classroom^49^ questionnaire. Perceived competence to deliver MI will be captured using an adapted version of the School Physical Activity Promotion Competence Questionnaire.^49^ The version to be used in the current study defines competence as ‘having the required skills to perform the task effectively (ie, with limited setbacks and well enough to result in desired outcomes)’. The scale uses five items (eg, ‘Create opportunities for my students to safely participate in physical activity in my classroom’) with responses on an eight-point scale (eg, 0=I have no skills in this area, 1–2=I have few skills in this area, 3–4=I have some skills in this area, 5=I have enough skills in this area to be competent, 6–7=I have many skills in this area).^49^

#### Pupil

##### Demographic

At measurement 0, each pupil’s name, date of birth, ethnicity, eligibility for free school meals and home postcode will be collected from school records. Home postcode will be used to calculate the English Index of Multiple Deprivation (IMD).

##### Anthropometric

At measurement 0, pupils’ height will be measured to the nearest 0.1 cm using a freestanding portable stadiometer (Seca, Leicester, UK) and weight will be measured to the nearest 0.1 kg using electronic weighing scales (Seca 899, Seca). Repeat measurements will be performed for each variable with the second measurement required to be within 0.4 cm or 0.1 kg; otherwise, a third measurement will be taken.^50^ Body mass index (kg/m^2^) will be calculated and expressed as a centile using the British 1990 growth reference data,^51^ with population monitoring body mass index cut-points applied to categorise weight status.^50^

##### Objective physical activity and sedentary time

At measurement 0 and measurement 3, pupils will be asked to wear an Actigraph accelerometer (GT3X or GT3X+) over the right hip using an elasticated waist band for seven consecutive days during waking hours. Pupils will be instructed to remove the devices when sleeping or during water-based activities to limit discomfort and potential device damage. These generations of Actigraph accelerometer display good accuracy and precision as an objective measure of physical activity and time spent sedentary.^52 53^

The following variables will be derived from the accelerometer data using the Evenson intensity cut-points^54^:

1. Mean minutes of MVPA (school day (eg, 09:00–15:15), total day and weekday and weekend day).
2. Mean minutes of light intensity physical activity (school day, total day and weekday and weekend day).
3. Mean minutes of sedentary time (school day, total day and weekday and weekend day).
4. Mean total volume of activity per day (counts per wear minute; school day, total day and weekday and weekend day).

##### Self-reported sedentary behaviour

At measurement 0 and measurement 3, self-reported weekday and weekend sedentary behaviours (outside of school) will be captured using an adapted version of the Adolescent Sedentary Activity Questionnaire.^55^ Pupils will report the duration of time they engage in a variety of sedentary behaviours (eg, using the computer for fun, watching TV, sitting around with friends and so on) in their free time on a typical weekday and weekend day.

##### Classroom engagement

Pupils’ engagement in the classroom will be assessed at measurement 0 and measurement 3, using the pupil self-report Engagement versus Disaffection with Learning scale.^56^ The scale includes 27 items rated on a 4-point Likert-type scale to measure emotional engagement, emotional disaffection, behavioural engagement and passive behavioural disaffection.^56^

##### Cognitive functioning

At measurement 0 and measurement 3, pupils’ visual selective attention, information processing speed and ability to concentrate will be evaluated using the d2 test.^57 58^ The d2, a cancellation task, consists of 14 lines each containing 48 characters and 658 total items. The task requires individuals to review the contents of each line of letters and to mark all ‘d’s with two dashes’. Twenty seconds per line are allowed, and the total administration time is approximately 6 min.^57 58^ The test will be administered via paper and pencil to the whole class simultaneously.

## Analyses​

Descriptive statistics will be used to describe the characteristics of the study population. A mixed methods approach will be used to explore implementation based on a convergent parallel design in which qualitative and quantitative data will be collected in parallel, analysed separately and then merged. This will permit an appraisal of the extent to which the two data streams converge, and the combination, which will allow for a more holistic understanding of the implementation of the CLASS PAL intervention.

Study objective A: the purpose of the analysis will be to determine the quantity and quality of implementation of CLASS PAL. Descriptive analyses will be used to present the degree of implementation (obtained from logs and teacher self-report items) over time and also between subgroups. Details on the type of activity delivered, supporting resources used, instruction type, duration of the activity and the pupils’ response will be quantitatively described using appropriate summary statistics (eg, frequency and percentage for categorical and binary variables, mean and SD for normally distributed continuous variables, and median and IQR for non-normally distributed continuous variables). The direct observation data will be presented to provide additional contextual information regarding on-task behaviour (before, during and after) and intensity or duration of the MI activities. The CLASS PAL website analytics will be documented over time, and comparisons with the quantity of implementation will be presented.

Quality of implementation will be ascertained from interview data with teachers and pupils as described below.

Study objective B: the purpose of the analysis will be to identify the processes by which teachers and schools implement CLASS PAL. Qualitative interview data from teachers and focus group data from pupils will be transcribed verbatim and coded using thematic analysis^59^ to determine the acceptability of CLASS PAL and MI more generally, as well as any barriers and facilitators to implementation. Although the interviews will be guided by a focused set of a priori topics, codes will primarily be drawn from the data and formed inductively.

Study objective C: the purpose of the analysis will be to investigate the individual (pupil and teacher) level and school-level characteristics associated with the quantity of implementation of CLASS PAL. Quantity of implementation (via the logs) will be descriptively presented using appropriate summary statistics for different subgroups (eg, high/low school-level support for physical activity; classroom environment (small/large) and so on). More distal pupil level indicators of implementation will be ascertained through school day accelerometer data.

First, to examine if the level of MI implementation is associated with school day physical activity, a linear multilevel model will be used with school day physical activity (via accelerometer) as the outcome variable, levels to indicate the clustering of pupils within class and within schools and the repeated measurement of physical activity, and a categorical indicator for degree of MI (from logs) as the explanatory variable. Second, to examine factors associated with implementation, potential mediating (eg, perceived competence to deliver MI), moderating (eg, classroom size and classroom engagement) and a priori confounder (eg, IMD) variables will be included.

## Discussion

This paper has outlined the protocol for a study to investigate the implementation of a MI intervention (CLASS PAL) in UK primary schools. Although there are some multistrategy approaches (eg, Transform-Us!),^29 30^ many previous interventions have focused on targeting a single type of strategy,^19 21 60^ as part of a prescribed approach, and thus have low plasticity. CLASS PAL provides a more plastic intervention approach, whereby teachers can use MI strategies that fit the particular needs of their class at a given moment. Furthermore, reviews of MI interventions have identified a paucity of evidence on the level of implementation achieved and what factors contribute to successful (or otherwise) implementation.^18 61^ The present study’s focus on evaluating the implementation of CLASS PAL and its influencing factors will therefore contribute towards closing a notable gap in the literature.

### Strengths and limitations

The strengths of this study include its pragmatic nature, the recruitment strategy to increase representation, frequency of interim measurements (three) and the mixed methods employed to garner in-depth detail on the degree of real-world implementation. Due to fiscal restraints and the desire to have a reasonable representation of one age group, this evaluation only included one school year group (year 5), which may limit the applicability of the findings across the entire primary school setting and particularly the lower years.

### Ethical considerations and dissemination

Participating class teachers will be required to return written consent to participate in the study, and they will be provided with a letter and information sheet. Children in the participating classes will be provided with a letter to parent(s)/guardian(s), parent/guardian information sheet and an age-appropriate child information sheet. Parents/guardians will be asked to complete an opt-out consent form only if they do not want their child to participate in the evaluation activities. This form of consent has been used in previous school-based physical activity interventions in the UK.^62^ Participating children will also be asked to sign an assent form prior to the first data collection session. Participants will be identifiable only by participant number and will have the right to withdraw from the study without any negative consequences at any time. If protocol alterations are required, permission will be sought from the University Human Participants Ethics Sub-Committee, participants notified, and any amendments reported in consequent publications.

Practical implications of findings are crucial to implementation research. Beyond academic conference presentation and peer-reviewed publication, the findings of this study will be disseminated to relevant school stakeholders through a number of regional and national avenues. These include regional Teaching School Alliances, regional and national print and online media, a growing social media presence (Twitter) and a 1-day symposium to be held at Loughborough University and to which all relevant stakeholder sectors and policy makers (eg, Public Health England, Ofsted and Department for Education) will be invited.

## Supplementary Material

Reviewer comments

## Footnotes

i http://www.affinitytsa.co.uk; Teaching School Alliances are groups of outstanding schools that work with others to provide high-quality training and development to new and experienced schools/school staff.

ii Personal Communication with management staff at Affinity Teaching School Alliance.

iii The Youth Sport Trust are a national school physical activity/wellbeing programme provider and charity - https://www.youthsporttrust.org
