# Supplementary material for: Study design and protocol for a mixed methods evaluation of an intervention to reduce and break up sitting time in primary school classrooms in the UK: The CLASS PAL (Physically Active Learning) Programme
Source: BMJ Open. 2017 Nov 8;7(11):e019428. doi: 10.1136/bmjopen-2017-019428 (PMC5695437; doi:10.1136/bmjopen-2017-019428)
Supplement: Reviewer comments [file bmjopen-2017-019428.reviewer_comments.pdf]

PEER REVIEW HISTORY

BMJ Open publishes all reviews undertaken for accepted manuscripts. Reviewers are asked to complete a checklist review form (**http://bmjopen.bmj.com/site/about/resources/checklist.pdf**) and are provided with free text boxes to elaborate on their assessment. These free text comments are reproduced below.

ARTICLE DETAILS

**TITLE (PROVISIONAL)**	Study design and protocol for a mixed methods evaluation of an intervention to reduce and break-up sitting time in primary school classrooms in the UK: The CLASS PAL (Physically Active Learning) Programme
**AUTHORS**	Routen, Ash; Biddle, Stuart; Bodicoat, Danielle; Cale, Lorraine; Clemes, Stacy; Edwardson, Charlotte; Glazebrook, Cristine; Harrington, Deirdre; Khunti, Kamlesh; Pearson, Natalie; Salmon, Jo; Sherar, Lauren

This article was not externally reviewed at BMJ Open. Protocols that have been independently assessed prior to submission to BMJ Open are usually fast-tracked to publication on the grounds that further substantial changes will not be possible. This independent assessment will usually be external, independent review for both a substantial grant award from a non-commercial or government funder and ethics approval.
